# Feasibility and acceptance of cervicovaginal self-sampling within the German National Cohort (Pretest 2)

**DOI:** 10.1007/s00103-014-2054-9

**Published:** 2014-10-11

**Authors:** Stefanie Castell, G. Krause, M. Schmitt, M. Pawlita, Y. Deleré, N. Obi, D. Flesch-Janys, Y. Kemmling, A.M. Kaufmann

**Affiliations:** 1 Department for Epidemiology, Helmholtz Centre for Infection Research, Inhoffenstraße 7, 38124 Braunschweig, Germany; 2 Hanover Medical School, Hanover, Germany; 3 Division of Genome Modifications and Carcinogenesis, Research Program Infection and Cancer, German Cancer Research Center, Heidelberg, Germany; 4 Department for Infectious Disease Epidemiology, Robert Koch-Institute, Berlin, Germany; 5 Department of Cancer Epidemiology, Clinical Cancer Registry, University Cancer Center Hamburg, University Medical Center Hamburg-Eppendorf, Hamburg, Germany; 6 Clinic for Gynecology, Charité – Universitätsmedizin Berlin, Berlin, Germany

**Keywords:** Self-sampling, Acceptability, German National Cohort (GNC), Cervicovaginal lavage, Human papillomavirus (HPV), Selbstbeprobung, Akzeptanz, Nationale Kohorte (NAKO), Cervicovaginale Lavage, Humane Papillomviren (HPV)

## Abstract

**Background and objectives:**

Within the German National Cohort (GNC) 100,000 adult women in Germany will be comprehensively interviewed and examined. While women’s health is addressed in the basic interview, direct detection of cervicovaginal microbial colonisation or infection is not part of the examination protocol. In a pilot project the feasibility of female study participants of the GNC collecting a cervicovaginal lavage at home without having to involve a gynecologist or other medical personnel was thus investigated. The ability of the procedure to detect vaginal microbes and conditions including human papillomavirus (HPV), *Chlamydia trachomatis* and bacterial vaginosis (BV) were also explored.

**Methods:**

This cross-sectional study was conducted in two study centers (Hamburg and Hanover) of the GNC during Pretest 2 in 2012 as an add-on module to the main program of the National Cohort. Participants were randomly selected through the population registration office. After providing written informed consent at the study center, participants self-collected a cervicovaginal lavage (Delphi Screener™) at home following written instructions. Participants mailed samples and acceptability questionnaires to the laboratory and the study center, respectively. Acceptability of self-sampling was categorized as consent, partial consent and rejection. The samples were analyzed by multiplex HPV genotyping for the presence of 27 mucosal HPV subtypes. To detect other pathogens “Sexually Transmitted Infection Profiling” (STIP) was used, a novel multiplex polymerase chain reaction (PCR) for various vaginally occurring pathogens/conditions coupled with subsequent bead-based Luminex^®^ hybridization. Human beta-globin and DNA polymerase alpha (PolA) sequences were used as positive controls for the detection of human DNA during HPV detection and STIP, respectively.

**Results:**

The participation based on the proportion of all women in Pretest 2 who could take part in the add-on Pretest 2 was 67.3 % (109 out of 162). The age of participants ranged from 20 to 69 years. The self-reported median duration of the collection of the lavage was 5 min. Analysis of the questionnaires (*n* = 108) revealed that the self-sampling of a cervicovaginal lavage was acceptable to 98 % of women (106 out of 108), and considered to be easy by 89 % (96 out of 108) as well as user-friendly by 96 % of the women (104 out of 108). Human beta-globin and PolA as markers for human DNA and sample quality were detected in all samples analyzed while HPV as a marker for pathogen detectability was identified in 18 out of 109 samples. Of the 107 samples tested with STIP as a second marker for pathogen detectability, 5 samples were excluded from statistical analyses on bacterial colonization because of signs in the laboratory results of the use of antibiotics. For the computation of the possible occurrence of bacterial vaginosis and candidiasis 7 and 8 samples, respectively, were excluded because of low signal intensities resulting in an evaluation of 95 or 94 samples, respectively. *Ureaplasma parvum* was detected in 22 out of 102 samples, BV in 14 out of 95 samples and candidiasis in 13 out of 94 samples. *Chlamydia trachomatis* was not detected in any sample.

**Conclusion:**

The feasibility study on cervicovaginal self-sampling indicates that this form of biosampling was very well accepted within the framework of the GNC and feasible in terms of pathogen detection. Its further application in the GNC would allow investigation of transience and persistence, or long-term effects of vaginal (co)infections and colonization.

## Abstract (de)

**Hintergrund und Ziele:**

In der Nationalen Kohorte (NAKO) werden 100.000 erwachsene Frauen in Deutschland umfassend befragt und untersucht werden. Während Frauengesundheit im Basisinterview thematisiert wird, ist der direkte Erregernachweis von cervicovaginalen mikrobiellen Besiedlungen oder Infektionen nicht Teil des Untersuchungsprotokolls. In einem Pilotprojekt untersuchten wir daher die Machbarkeit der häuslichen Selbstentnahme einer cervicovaginalen Lavage durch Studienteilnehmerinnen der NAKO ohne Kontakt zu einem Frauenarzt oder anderem medizinischen Personal. Wir explorierten außerdem die Möglichkeit bei diesem Vorgehen, verschiedene vaginal auftretende Erreger und Erkrankungen, inklusive humane Papillomviren (HPV), *Chlamydia trachomatis* und bakterielle Vaginose (BV) nachzuweisen.

**Methoden:**

Wir führten die Studie als Querschnittsstudie 2012 während Pretest 2 als zusätzliches Modul zum Hauptprogramm der NAKO in zwei Studienzentren (Hamburg und Hannover) durch. Die Teilnehmerinnen entstammen einer zufällig gezogenen Einwohnermeldeamtstichprobe. Sie führten nach Aufklärung und schriftlicher Einverständniserklärung im Studienzentrum auf Basis einer schriftlichen Anweisung die Probenentnahme zuhause durch (Delphi Screener^TM^). Die Proben und Akzeptanzfragebögen wurden per Post von den Teilnehmerinnen direkt an das Labor bzw. an das jeweilige Studienzentrum gesandt. Akzeptanz wurde in drei Kategorien abgefragt: Zustimmung, teilweise Zustimmung, Ablehnung. Die Proben wurden per Multiplex-Papillomvirusgenotypisierung auf Anwesenheit von 27 mukosalen HPV-Typen untersucht. Der Nachweis anderer Pathogene erfolgte durch „Sexually Transmitted Infection Profiling“ (STIP), einer neuen Multiplex- Polymerase-Kettenreaktion (PCR) für verschiedene vaginal auftretende Erreger/Erkrankungen mit anschließender „Bead“-basierter Luminex^®^-Hybridisierung. Als Positivkontrolle wurden ß-Globin (HPV-Nachweis) und die humane DNA Polymerase alpha (PolA)-Sequenz (STIP) nachgewiesen.

**Ergebnisse:**

Die Teilnahme als Anteil an allen Frauen in Pretest 2, die an Zusatzmodulen in den betreffenden Studienzentren teilnehmen konnten, lag bei 67,3 % (109/162). Die Altersspanne der Probandinnen reichte von 20 bis 69 Jahre. Im Median dauerte die Probenentnahme nach Angaben der Probandinnen 5 Minuten. Die Analyse der Akzeptanzfragebögen (*n* = 108) ergab, dass die selbstständige Durchführung einer cervicovaginalen Lavage für 98 % der Frauen akzeptabel war (106/108), dass 89 % die Probenentnahme als leicht (96/108) und 96 % als benutzerfreundlich einstuften (104/108). Beta-Globin und PolA als Marker für humane DNA und Probenqualität konnte bei allen untersuchten Proben nachgewiesen werden, während HPV als Marker für Pathogenerfassbarkeit in 18 von 109 Proben nachgewiesen wurde. Von 107 mittels STIP als zweitem Marker für Pathogenerfassbarkeit untersuchten Proben wurden 5 von den statistischen Analysen bakterieller Erreger aufgrund von Hinweisen in den Laboranalysen auf Antibiotikaeinnahme ausgeschlossen. Um zu berechnen, ob bakterielle Vaginose oder Candidose vorhanden sind, wurden 7 bzw. 8 Proben aufgrund niedriger Signalintensitäten ausgeschlossen. Dies führte zur Evaluierung von 95 bzw. 94 Proben. Wir konnten z.B. *Ureaplasma parvum* in 22 von 102 Proben nachweisen, bakterielle Vaginose in 14 von 95 Proben und Candidose in 13 von 94 Proben. *Chlamydia trachomatis* konnte in keiner Probe detektiert werden.

**Schlussfolgerung:**

Unsere Machbarkeitsstudie zur cervicovaginalen Probenselbstentnahme zeigt, dass diese Bioprobengewinnung im Kontext der NAKO sehr gut akzeptiert wurde und hinsichtlich Pathogennachweisen machbar ist. Die zukünftige Anwendung in der Nationalen Kohorte würde es ermöglichen, Transienz/Persistenz oder langfristige Effekte vaginaler (Ko-) Infektionen und Kolonisationen zu untersuchen.

Gynecological infections, such as bacterial vaginosis (BV) are frequent [1] and carry a high psychological burden especially if recurrent [2] and infections with human papillomavirus (HPV) may have serious consequences, such as cervical carcinoma [3]. Epidemiological studies investigating such conditions should include cervicovaginal biosampling due to limitations of self-reported medical history and serological detection [e.g. 4]; however, in population-based general cohort studies that depend on long-term follow-up like the German National Cohort (GNC), any extra burden on participants has to be kept to a minimum. Hence, additional modules on infectious diseases should implement biosampling methods that can be conducted easily and preferably at home as self-sampling in order to minimize any additional time spent at the study center.

Most studies in a systematic review conducted in 2007 showed that self-sampling of vaginal biomaterial for detection of HPV deoxyribonucleic acid (DNA) using a variety of techniques and tools was feasible, acceptable and adequate for analysis [5]. A more recent systematic review on the same topic from 2010 reaffirmed the finding that self-sampling is generally well perceived and easy for women to perform; however, the authors emphasized that methodological limitations, such as measurement error and selection bias could result in an overestimation of these findings [6]; but self-sampling is applicable even in low-resource settings [7]. For Germany, Deleré et al. demonstrated that self-collection of cervicovaginal lavage is a valid approach for detection of HPV [8] and was also successfully used in a population-based study in young female adults in Germany [9].

Adding cervicovaginal lavage to the investigations in the GNC would open unprecedented opportunities to prospectively investigate associations between cervicovaginal infection and colonization with a comprehensive phenotyping of non-infectious diseases; however, given the age distribution of the GNC and the extensive investigations on the participants (see Ahrens et al. in this issue), it is not clear to what extent such a self-collection approach is feasible within the GNC. This study therefore aimed to examine the feasibility and acceptance of self-sampling of cervicovaginal lavage within the framework of the GNC taking into account the biological quality of the samples and applicability of a new high-throughput diagnostic approach developed by Schmitt et al. [10]. Additionally, the results of all pathogen detections were to be evaluated considering the results in the literature from similar studies.

## Methods

### Study design, population and self-sampling

This cross-sectional study took place in two study centers (Hamburg and Hanover) of the GNC during Pretest 2 (September 2012 to December 2012) (see Ahrens et al. in this issue) as an add-on module to the main program, which is described by Wichmann et al. [11]. Briefly, 18 study centers in Germany will interview and examine 200,000 men and women randomly selected from the population register. The examinations and interviews cover cardiovascular, metabolic, neurological and infectious diseases. In concordance with the GNC, the age structure of the study population in Pretest 2 was aimed at 10 % for 20–29 and 30–39-year-olds and 26.7 % for each of the age groups 40–49, 50–59 and 60–69 years [11].

The analysis of this feasibility study is limited to Pretest 2 participants randomly sampled through the population registration office. Study participation was offered to all female participants recruited for Pretest 2 (*n* = 162) and accessible for add-on modules. Pregnant women and women within 30 days after childbirth were excluded. Informed consent for this additional study on collecting a cervicovaginal lavage was obtained during the participant’s visit to the study center. After receiving the sampling kit at the study center, self-sampling was done at home. The tool applied (second generation Delphi Screener^TM^, Delphi Bioscience, BV Scherpenzeel, The Netherlands) is a sterile instrument with 3 ml of physiological saline. After inserting the device into the vagina, followed by pushing a button at the end of the Screener, the lavage liquid is released into the vagina (near the cervix). The fluid is automatically reabsorbed by the device during removal from the vagina after the button is released. The participants then transferred the lavage into a transportation tube and mailed it to the laboratory (Charité, Berlin) where the HPV status of the samples was determined. Results were regularly reported back to the participants. After completion of recruitment, testing for further microbes was done at the German Cancer Research Center, Heidelberg, using sexually transmitted infection profiling (STIP). Participants whose samples indicated the presence of *Neisseria gonorrhoeae* infection were recommended to immediately contact a gynecologist for confirmation by clinical diagnosis. If sample analysis by STIP resulted in any other suspicious findings participants received a recommendation to see their gynecologist.

### Questionnaire on acceptability

The questionnaire contained a question on sexual history (ever had sexual intercourse: yes/no) and assessed the acceptability of sampling as consent (i.e. yes/partly/no) to the following statements: “The acquisition of a vaginal lavage at home was acceptable for me.”, “It was easy to self-sample the specimen.”, “The sampling was user-friendly.”, “The sampling was physically uncomfortable.”, “I consider sampling of a vaginal lavage a violation of privacy.” and “It is more acceptable to sample a vaginal lavage at home than at a study center.”. Demographic data and medical history were collected during the main interview of the GNC.

### DNA extraction and HPV genotyping

Total DNA was extracted using the Qiagen DNA mini kit (Hilden, Germany) and a broad spectrum GP5 + /GP6 + -based polymerase chain reaction (PCR) including beta-globin was conducted as described [12] followed by Luminex^®^-based HPV genotyping (multiplex HPV genotyping, Luminex, Austin TX [13]). For DNA sample validation a PCR for human beta-globin DNA was performed and products were separated and read on 2 % agarose gels. Only samples showing a specific product were considered to be DNA positive. The detection of HPV 6, 11, 16, 18, 26, 31, 33, 35, 39, 42, 43, 45, 51, 52, 53, 54, 56, 57, 58, 59, 66, 68, 70, 72, 73, 82 and 90 was carried out using self-produced multiplex master mixes.

### Sexually transmitted infection profiling (STIP)

The STIP was carried out as previously described [10]*.* Briefly, STIP is a multiplex PCR for microbial target sequences followed by hybridization of the biotinylated amplimers to specific probes on fluorescent beads (xMAP technology, Luminex^®^). The STIP is able to simultaneously identify *Atopobium vaginae, Candida albicans, C. glabrata, C. krusei, Chlamydia trachomatis*, *Gardnerella vaginalis,* herpes simplex virus (HSV) 1, HSV 2, *Mycoplasma genitalium*, *M. hominis*, *M. pneumoniae, M. spermatophilum*, *Neisseria gonorrhoeae*, *Treponema pallidum*, *T* *richomonas vaginalis*, *Ureaplasma urealyticum* and *U. parvum*, and the bacteria of the normal genital flora *Lactobac* *i* *llus iners*. Other *Lactobacillus* species, including *L. gasseri*, *L. crispaticus*, *L. jensenii* and *L. vaginalis* were detected together by a universal *Lactobacillus* probe. Additional *Candida* species, e.g. *C. parapsilosis* and *C. tropicalis*, were detected by a universal *Candida* probe. Presence of amplifiable human DNA was verified using the human DNA polymerase alpha (PolA) sequence as a target. The load of *A. vaginae, G. vaginalis* and *Lactobacillus* species was determined and a BV score using load ratios of either *A. vaginae* or *G. vaginalis* over *Lactobacillus* was calculated as previously described [10]. The score grades for BV were as follows: 0 absence of BV, 1 weak indication for BV, 2 some indications for BV, 3–4 strong indications for BV and 5 very strong indications for BV. Likewise, candidiasis was determined by a ratio of signals from several *Candida* spp. probes and *Lactobacillus* as previously described [10]. In cases where signal intensities of pathogens and *Lactobacillus* were too low, BV and candidiasis could not be computed.

### Definitions and statistical analysis

Migration status was defined according to Schenk et al. [14] as either both parents not born in Germany or one parent not born in Germany and interviewee not living in Germany since birth or German not being native language. Household net equivalent income per month was calculated using midpoint estimates of group levels; the highest group (≥ 8000 €) was set at 10,000 €. To account for household size, weighting was done according to the German demographic standards [15] using the modified Organisation for Economic Co-operation and Development (OECD) equivalence scale. Women were categorized according to their menstruation status as premenopausal versus perimenopausal/postmenopausal. For bacteria detected by STIP, a participant was included into the statistical analysis if at least one species of microbe was detected and amplified human DNA (PolA) was present. For samples without detection of any microbial agent, potential treatment with antibiotics was assumed [10] and these specimens were therefore excluded from the respective statistical analysis (bacteria). For the analysis regarding viruses, all samples were included as viruses are not affected by antibiotics. The age of participants was grouped in 10-year increments (e.g. 20–29 and 30–39 years). To test differences between groups for statistical significance (comparisons of participants and non-participants, of age groups and categories of acceptability, and of time elapsed until arrival of samples at the laboratory depending on HPV status), for nominal or ordinal variables *χ* ^2^-test and Fisher’s exact test were used and for continuous variables the Mann-Whitney test after evaluating observed data regarding normal distribution using graphical tools and the Shapiro-Wilk test. All statistical tests were 2-sided and a *p*-value < 0.05 was considered statistically significant. Proportions were calculated with missing or unspecified values as an additional category, whereas statistical tests were performed without this category. Approximate binomial confidence intervals (CI) were calculated based on the formula given in [16] using Excel (Microsoft, Redmond WA). For all other statistical analyses STATA 12 (StataCorp LP, College Station TX) was used.

## Results

### Participation

**Fig. 1:**
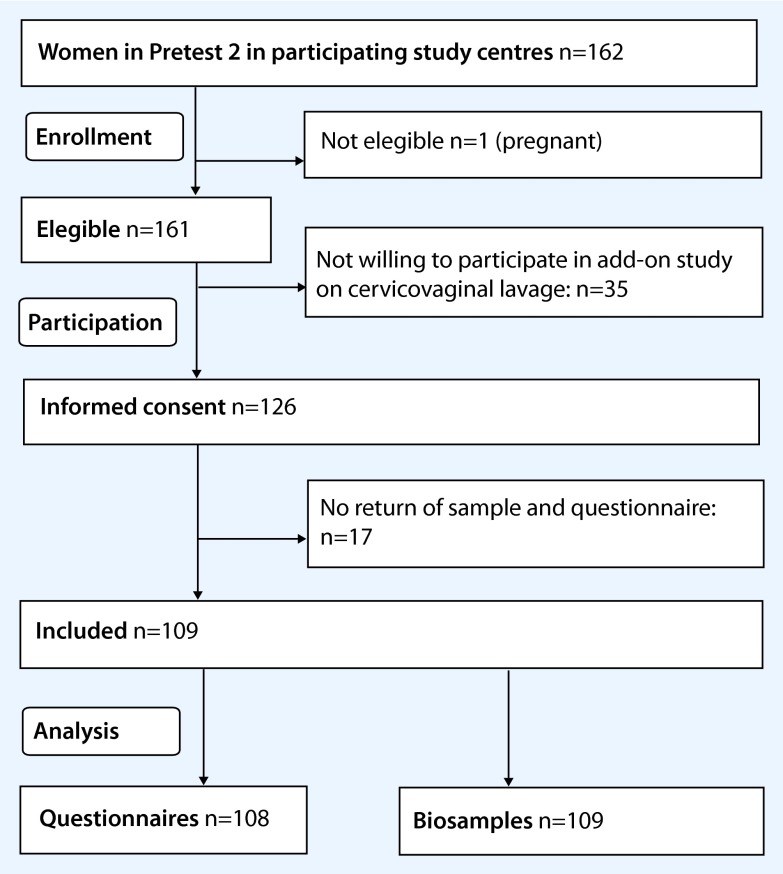
Flow chart of participation in the study on cervicovaginal lavage

Of the 162 women initially recruited for Pretest 2 from a random registration office sample, 1 woman was excluded due to pregnancy and 126 (77.8 %) agreed to participate in the additional module on cervicovaginal lavage, from whom 109 (86.5 %) samples and 108 (85.7 %) completed questionnaires were received (Fig. 1). The participation in the add-on module within Pretest 2 of the GNC was thus 67.3 % (109 out of 162, 95 % CI 59.7; 74.0).

Characteristics of participating and non-participating women are presented in Table 1. A significant difference between both groups was detected with respect to hysterectomy. The median age of participating women was 5 years lower than that of non-participating women (no significant difference) (Table 1).

**Table 1:** Characteristics of participants^a^ versus women with no participation^b^

Variable	Category	Total	Participation	No participation	*P-*value*
		*n* = 161	*n* = 109	*n* = 52	
Age (years)	Median (min; max)	53 (20; 69)	51 (20; 69)	56 (21; 69)	0.06
Age groups *n* (%)	20–29 years	11 (6.8)	7 (6.4)	4 (7.7)	0.33
	30–39 years	18 (11.2)	15 (13.8)	3 (5.8)	
	40–49 years	37 (23.0)	28 (25.7)	9 (17.3)	
	50–59 years	49 (30.4)	31 (28.4)	18 (34.6)	
	60–69 years	46 (28.6)	28 (25.7)	18 (34.6)	
Migration status *n* (%)	No	135 (83.9)	89 (81.7)	46 (88.5)	0.17
	Yes	25 (15.5)	20 (18.3)	5 (9.6)	
	Not specified	1 (0.6)	0	1 (1.9)	
Household net equivalent income *n* (%)	≤ 1500€	45 (28.0)	30 (27.5)	15 (28.8)	0.82
	1500 < x ≤ 3000€	77 (47.8)	51 (46.8)	26 (50.0)	
	> 3000€	39 (24.2)	28 (25.7)	11 (21.2)	
Menstruation *n* (%)	Premenopausal	50 (31.1)	38 (34.9)	12 (23.1)	0.13
	Perimenopausal/postmenopausal	111 (68.9)	71 (65.1)	40 (76.9)	
Hysterectomy *n* (%)	No	128 (79.5)	93 (85.3)	35 (67.3)	0.008
	Yes	33 (20.5)	16 (14.7)	17 (32.7)	

^a^Mailed questionnaire and biosample or biosample only

^b^Did not give informed consent for the study or did not return the questionnaire and/or biosample

*χ ^2^-test or Fisher’s exact test, except for age as continuous variable (Mann-Whitney test)

### Timing

The median time from the visit at the study center to sampling was 7 days (*n* = 107, interquartile range (IQR) 3–16 days, range 0–90 days). Median reported duration of sampling was 5 min (n = 105, IQR 3–6 min, range 1–30 min). It took a median time of 2 days (*n* = 102, IQR 2–4 days, range 1–140 days) after self-sampling for the specimens to arrive at the laboratory. There was no significant difference in this respect between HPV positive and negative samples (identical median, *p* = 0.13).

### Acceptance

**Fig. 2:**
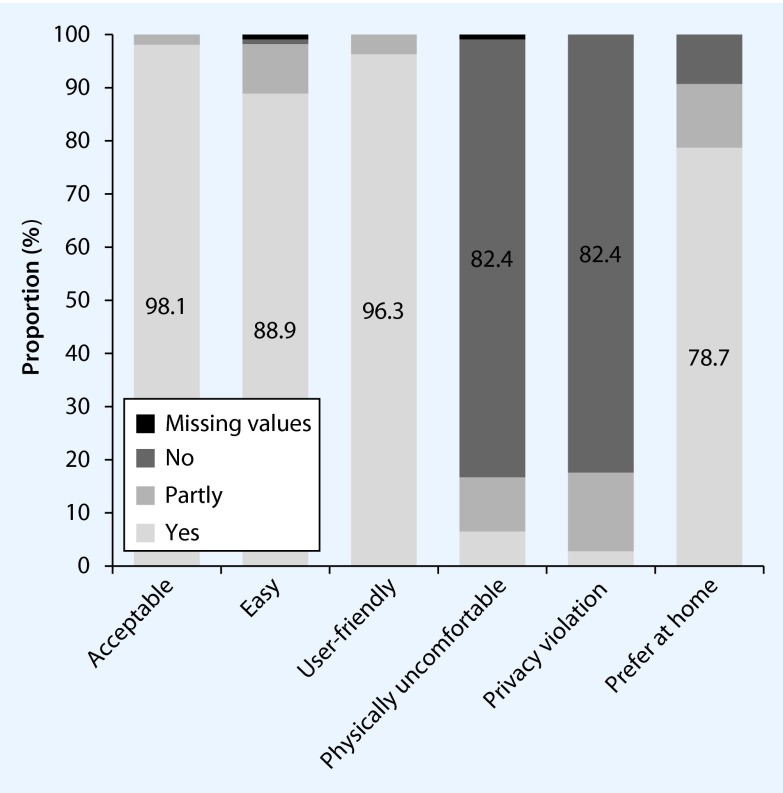
Assessment of acceptability of procedure and device used for biosampling (*n* = 108)

A total of 98.1 % (106/108, 95 % CI 93.5; 99.5) judged the sampling of a cervicovaginal lavage at home to be acceptable, 88.9 % (96/108, 95 % CI 81.6; 93.5) as easy and 96.3 % (104/108, 95 % CI 90.9; 98.6) as user-friendly. Only 6.5 % reported a physically uncomfortable feeling during sampling (7/108, 95 % CI 3.2; 12.8) and 10.2 % (11/108, 95 % CI 5.8; 17.3) stated “partly” for this item. Self-sampling of a cervicovaginal lavage was seen as no violation of privacy by 82.4 % (89/108, 95 % CI 74.2; 88.4) and was preferably done at home instead of at the study center in 78.7 % of cases (85/108, 95 % CI 70.1; 85.4) (Fig. 2).

No statistically significant differences could be detected between age groups and any of the aforementioned categories of acceptability with the exception of the item on privacy violation (here *p* = 0.02, *n* = 108); other results of Fisher’s exact test for comparison of each category and age groups: acceptance *p* = 0.2 (*n* = 108), easiness *p* = 0.86 (*n* = 107), user-friendliness *p* = 0.87 (*n* = 108), physical discomfort *p* = 0.26 (*n* = 107) and preferred place of sampling *p* = 0.63 (*n* = 108).

With respect to age categories, sampling a cervicovaginal lavage was viewed as no violation of privacy by 57.1 % (4/7) of 20–29-year-olds, by 60.0 % (9/15) of 30–39-year-olds, 82.1 % (23/28) of 40–49-year-olds, 87.1 % (27/31) of 50–59-year-olds and 96.3 % (26/27) of 60–69-year-olds.

Two minor adverse events without the necessity to contact a doctor were reported (i.e. slightly painful sensation in lower abdomen after sampling). Three women aged between 32 and 65 years stated no previous sexual intercourse; all of whom assessed the sampling as acceptable and user-friendly, 2 as easy, physically not uncomfortable and as no violation of privacy.

Human beta-globin DNA was detected in all samples (*n* = 109) and HPV DNA was identified in 18 out of 109 samples: 10 different genotypes were found (HPV11, 16, 26, 31, 39, 42, 54, 57, 66, and 68) and 6 out of 18 HPV positive women were infected with at least 2 genotypes. HPV16 was found in 6 out of 18 positive samples and all other genotypes were detected less often.

Two samples arrived too late to be included in the STIP (*n* = 107). PolA DNA quality was sufficient for analysis of all samples analyzed by STIP; five samples were excluded from statistical analyses of bacterial occurrence because no microbes could be detected by STIP which was interpreted as potential antibiotic use. The bacteria *A. vaginae*, *U. parvum*, Candida spp., *C. albicans, G. vaginalis*, *M. genitalium, M. hominis*, *M. spermatophilum*, *C. krusei*, and *N. gonorrhoeae* were detected (Table 2). In the case of pathogen and *Lactobacillus* intensities being too low, BV (*n* = 7) and candidiasis (*n* = 8) could not be computed. Evidence for BV was present in 14 out of 95 of specimens, of which 9 had a BV score of 2 (i.e. some indications for BV) and 5 a score of 4–5 (strong and very strong indications for BV). Candidiasis was detected in 13 out of 94 samples and BV, *U. parvum;* and candidiasis occurred in all age groups (data not shown).

**Table 2:** Pathogens and conditions detected by sexually transmitted infection profiling *(STIP)*

Pathogen/condition	*n*
*n* = 107, all samples analyzed by STIP	
Herpes simplex virus 1	0
Herpes simplex virus 2	0
*n* = 102, exclusion of 5 samples due to signs of antibiotic use	
A. vaginae	22
C. trachomatis	0
G. vaginalis	15
M. genitalium	4
M. hominis	1
M. pneumoniae	0
M. spermatophilum	3
N. gonorrhoeae	1
T. pallidum	0
T. vaginalis	0
U. urealyticum	0
U. parvum	22
C. albicans	18
C. glabrata	0
C. krusei	2
Other Candida spp.	21
*n* = 95, additional exclusion of 7 samples (low signals)	
Bacterial vaginosis	14
*n* = 94, additional exclusion of 8 samples (low signals)	
Candidiasis	13

## Discussion

In this feasibility study it could be shown that participation in an add-on module on self-sampling of a cervicovaginal lavage within the GNC was sufficient to design a future study based on the tested approach for the main phase of the GNC. Acceptability of self-sampling at home was high among the female Pretest 2 participants in Hanover and Hamburg. The high acceptability of the device used for self-sampling is in line with a German study among 20–30-year-old women routinely screened by gynecologists in which participants reported the device as being easy to use and were fine regarding sensation [8]. In a population-based prevalence study among 20–25-year-old women conducted by Deleré et al. 73.4 % of the participants stated that self-sampling was easy or very easy [9]. In The Netherlands, the device has already successfully been used in a trial on cervical screening comparing self-sampling and regular screening by physicians [17]. Other devices for vaginal self-sampling, such as swabs have been effectively used in epidemiological and clinical studies (e.g. [18]), mainly within the context of cervical cancer screening [5].

In addition, it could be shown that self-sampling of a cervicovaginal lavage is also acceptable and applicable within the GNC, where women are typically older than in most previously cited studies and also already involved in an extensive protocol of epidemiological examinations. It is of note that willingness to participate was less likely among older women and among those with a hysterectomy. The latter might partly be a result of HPV infection being emphasized during some of the preconsent counselling and feedback of results, which may have been of less interest to women after hysterectomy. In future similar studies among older women, participants should be informed about the respective benefits of participation. With respect to acceptability, the only significant association with age was that younger women more frequently felt that their privacy during the self-sampling was affected than older women; however- overall, participants were still younger than non-participants. With respect to other effects of age on categories of acceptability, the power of this study might not be sufficient to determine any additional statistically significant differences. The fact that participants were younger than non-participants suggests that selection bias could be present in this study; therefore, the study results could overestimate acceptability.

The results of this study show that the chosen approach generates high quality biospecimens that allow molecular biological detection of various vaginal microbes. There were no statistically significant differences in the time elapsed until the biosamples arrived in the laboratory depending on HPV status; however, the statistical power of the study might not be sufficient to detect a difference. The most common genotype could be confirmed as HPV 16 [3, 9, 19] but less frequent HPV genotypes could also be detected. The detection of HPV and other colonizing agents was done (a) as an incentive for the women to participate, (b) to evaluate the quality of the biosamples and (c) to assess the study in comparison to similar studies. The small sample size does not therefore allow a thorough interpretation of the results concerning prevalence of HPV, BV, candidiasis and any of the other pathogens detected using STIP.

Deleré et al. used the same sampling device and HPV detection method and reported an HPV prevalence of 38.1 % among non-vaccinated 20–25-year-olds [9]. The age-specific estimate is similar (data not shown); however, the very small sample size of this age group considerably limits this comparison. The concordance between the studies could also be due to chance.

Due to the use of different tests with different numbers of genotypes, comparisons with other studies regarding non-genotype-specific prevalence are only suitable to a limited extent: For example, a German study by Klug et al. recruiting women over the age of 30 years within the context of routine cervical cancer screening, having specimens taken by physicians and using Hybrid Capture 2 as a screening test, reported an overall HPV prevalence of 5 % (30–39 years 6.9 % to 60 + years 2.5 %) which is lower than the results of this study [19]. This might be the case because the HPV detection method used in this study identified a greater number of HPV genotypes. In addition, the recruitment strategies in both studies differed (women participating in gynecological screening in Klug et al. [19] versus random sample from the registration office in this study) which might lead to diverse results. Women taking part in this study might have suspected a HPV infection potentially leading to selection bias and more HPV detection. In general, selection bias could distort estimates of positive testing in either direction in this study.

Within this add-on module the applicability of a new high-throughput diagnostic approach in the general population was also tested [10]. Detection of BV was only slightly lower than in other population-based studies in developed countries among Caucasian women (e.g. [1]).

*C. albicans* was the most common singly identified Candida species which is in line with reports from the literature [20]. *Chlamydia trachomatis,* like several other sexually transmitted pathogens, was not identified within this study population. This could be due to the small sample size but also due to the participants’ age being mostly over 40 years. Furthermore, some of these pathogens may be more prevalent in other parts of the genital tract because of the ascension to the upper genital tract and might therefore not be sampled with the device used in this study.

These results should be interpreted with caution as STIP is a newly developed method, albeit shown to be accurate and robust [10]. Furthermore, the number of analyzed biosamples was not designed to detect less common pathogens of the genital tract or calculate age-specific estimates so that all results regarding pathogens should only be interpreted as markers for detectability.

## Conclusions

This study shows that self-sampling of a cervicovaginal lavage at home independently of a gynecological examination is highly accepted among participants of the GNC and generates high quality specimens that allow nucleic acid amplification tests for pathogen detection. This opens a promising opportunity to study the occurrence of genital infection and colonization in the context of a comprehensive phenotyping of highly prevalent non-infectious diseases in a prospective and population-based cohort design. Given the public health importance of research questions resulting from this opportunity it is strongly recommended that self-sampling of cervicovaginal lavage is included as an additional module within the GNC.

### Acknowledgements

The authors would like to thank the staff at the study centers for their commitment, the study participants for making the study possible, U. Schiller for performing HPV tests, A. Schultze and M. Akmatov (Helmholtz Centre for Infection Research) for valuable discussion, C. Sievers and F. Pessler (TWINCORE Center for Experimental and Clinical Infection Research) for a critical reading of the manuscript, and R. Hol and M. Voll (Delphi Bioscience) for their support.
